# Spatial analysis of Chikungunya fever incidence and the associated socioeconomic, demographic, and vector infestation factors in municipalities of Pernambuco, Brazil, 2015–2021

**DOI:** 10.1590/1980-549720230018

**Published:** 2023-02-20

**Authors:** Maísa Aguiar-Santos, Liana Gabriele da Cruz Mendes, Diogenes Ferreira dos Passos, Tamyris Gomes da Silva Santos, Rosanny Holanda Freitas Benevides Lins, Ana Cristina Pedrosa do Monte

**Affiliations:** I Instituto de Medicina Integral Professor Fernando Figueira, Multiprofessional Residency Program in Collective Health – Recife (PE), Brazil.; II Secretaria de Saúde do Estado de Pernambuco, Environmental Surveillance – Recife (PE), Brazil.; III Fundação Oswaldo Cruz, Centro de Pesquisas Aggeu Magalhães, Graduate Program in Public Health – Recife (PE), Brazil.; IV Secretaria de Saúde do Estado de Pernambuco, Epidemiological Surveillance – Recife (PE), Brazil.; V Universidade Federal de Pernambuco, Centro de Ciências Médicas, Graduate Program in Tropical Medicine – Recife (PE), Brazil.

**Keywords:** Arbovirus infections, Aedes aegypti, Chikungunya virus, Spatial analysis, Socioeconomic factors, Public health surveillance, Infecções por Arbovirus, Aedes aegypti, Vírus Chikungunya, Análise espacial, Fatores socioeconômicos, Vigilância em saúde pública

## Abstract

**Objective:**

To identify the spatial patterns of chikungunya fever (CHIKF) and the associated socioeconomic, demographic, and vector infestation factors in the 1^st^ Health Region of Pernambuco (1^st^ HRP).

**Methods:**

This ecological study used a spatial analysis of Mean Incidence Rates (MIR) of probable cases of CHIKF reported among residents of the 19 municipalities of the 1^st^ HRP, in 2015–2021. The univariate and bivariate global Moran indexes (I) were estimated. From the significant associations (p<0.05), clusters were identified using the local Moran index and maps.

**Results:**

A predominance of the largest CHIKF rates was identified in the east. However, there was a heterogeneous distribution of rates across municipalities, which may have contributed to the absence of spatial autocorrelation of CHIKF (I=0.03; p=0.294) in univariate I. The bivariate I revealed a positive spatial correlation between CHIKF and the Municipal Human Development Index (MHDI) (I=0.245; p=0.038), but with a cluster of cities with low incidences and low MHDI in the west. There was no spatial correlation between CHIKF and the other variables analyzed: population density, Gini index, social vulnerability index, and building infestation index for *Aedes aegypti*.

**Conclusions:**

The results suggest that only the MHDI influenced the occurrence of CHIKF in the 1^st^ HRP, so that municipalities in the west demonstrated spatial dependence between lower values of MHDI and MIR. However, this spatial correlation may have occurred due to possible underreporting in the area. These findings can assist in the (re)orientation of resources for surveillance and health care services.

## RESUMO

**Objetivo:**

Identificar, na Iᵃ Região de Saúde de Pernambuco (Iᵃ RSP), os padrões espaciais da febre de Chikungunya (CHIKF) e os fatores socioeconômicos, demográficos e de infestação vetorial associados.

**Métodos:**

Este estudo ecológico utilizou a análise espacial das Taxas Médias de Incidência (TMI) de casos prováveis da CHIKF notificados entre os residentes dos 19 municípios da Iᵃ RSP no período de 2015–2021. Os índices de Moran global (I) univariados e bivariados foram estimados. Das associações significativas (p<0,05), *clusters* foram localizados por meio do Índice de Moran Local e de mapas.

**Resultados:**

Identificou-se predominância das maiores TMI da CHIKF no leste. Entretanto, houve distribuição heterogênea das taxas dos municípios, o que pode ter contribuído para a ausência de autocorrelação espacial da CHIKF (I=0,03; p=0,294) no I univariado. O I bivariado revelou correlação espacial positiva entre a CHIKF e o Índice de Desenvolvimento Humano Municipal (IDHM) (I=0,245; p=0,038), porém com um *cluster* de cidades com baixas incidências e baixo IDHM no oeste. Não houve correlação espacial entre a CHIKF e as demais variáveis analisadas: densidade demográfica, Índice de Gini, Índice de Vulnerabilidade Social e Índice de Infestação Predial de *Aedes aegypti*.

**Conclusões:**

Os resultados sugerem que somente o IDHM influenciou na ocorrência da CHIKF na Iᵃ RSP, de forma que municípios do oeste demonstraram dependência espacial entre menores valores de IDHM e TMI. No entanto, essa correlação espacial pode ter ocorrido devido às possíveis subnotificações na área. Tais achados podem auxiliar na (re)orientação de recursos dos serviços de vigilância e assistência à saúde.

## INTRODUCTION

Arboviruses transmitted by the *Aedes aegypti* mosquito are one of the greatest obstacles to Public Health worldwide, including Chikungunya fever (CHIKF), which reached 137,025 reported cases in the Americas in 2021^1^. Of these records, approximately 97% occurred in Brazil^1^, where the Pernambuco state stood out for the highest incidence rate of CHIKF (329 probable cases/100,000 inhabitants)^2^.

Autochthonous cases of CHIKF in Pernambuco were reported for the first time in 2015^3^. After that, the state experienced alternating between endemic and epidemic periods, with considerable increases in the years 2016, 2020, and 2021^2,4,5^. Of the 12 health regions into which the state is divided^6^, the 1^st^ Health Region of Pernambuco (1^st^ HRP) was responsible for 81.2% of all probable cases notified in the previous year (2021)^2^, which contributed to registering the highest incidence rate (600.5/100,000 residents) among regions.

Characterized by intense arthralgia and possible atypical manifestations (such as neurological manifestations) that can persist for years^7^, CHIKF has the potential to cause overload of health services and work absenteeism^7^. Both the magnitude and dissemination of this disease are influenced by the presence and abundance of the *A. aegypti* vector^8^, whose proliferation depends on socioeconomic, demographic, and environmental factors^8–10^. Studies show that urban areas with precarious sanitation conditions and high population density provide ideal breeding grounds for these vectors, favoring an increase in the incidence of arboviruses^10,11^. Spaces like this can be common in Pernambuco, a state that also stands out for having the highest poverty rate^12^ and for occupying 19^th^ place in the ranking of the Human Development Index (HDI) in the country^13^. However, there are contradictory findings in the literature^14–17^ that indicate both the presence of associations between high incidence of arboviruses and better socioeconomic conditions and the absence of any association.

In view of the above, it is essential to know which determinants interfere in the health-disease process of a territory, so that there is guidance for effective control and prevention actions. That said, spatial analysis techniques become extremely useful instruments, as they have been used to investigate the spatial dynamics of diseases such as arboviruses in the social context in different locations, addressing possible associated factors and detecting vulnerable areas^8,9,16–18^. However, there are gaps in the knowledge of these factors in the context of CHIKF in Pernambuco.

Therefore, the present study aimed to identify the spatial patterns of CHIKF and the associated socioeconomic, demographic, and vector infestation factors, in the municipalities of the 1^st^ HRP, in the period from 2015 to 2021. Thus, valuable directions can be indicated for Surveillance in Health.

## METHODS

This is an ecological study with spatial analysis referring to the incidence rates of probable cases of CHIKF in residents of the 1^st^ HRP, from 2015 to 2021, as described by Costa et al.^16^. The units of spatial analysis used were municipalities.

### Study area

**Figure 1:**
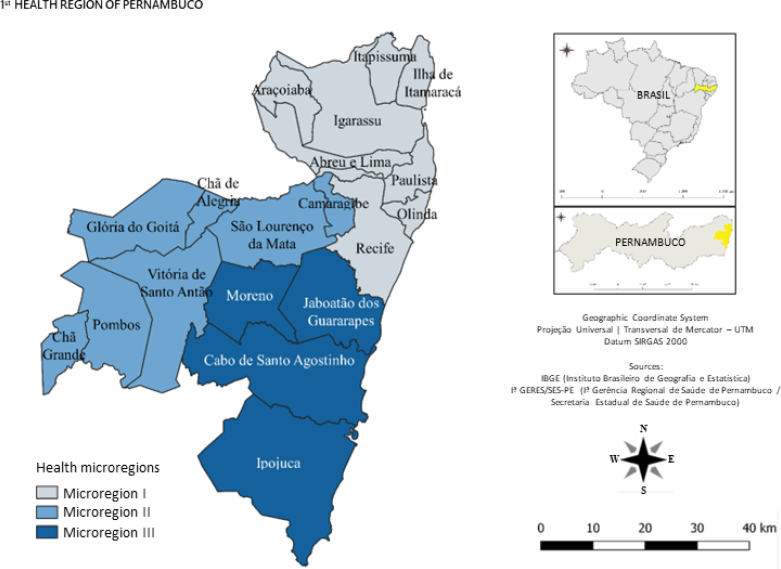
Map referring to the division of municipalities in the 1^st^ Health Region of Pernambuco, Brazil, into microregions.

Pernambuco is divided into 12 health regions, including the 1^st^ Region, which concentrates most of the state's resident population (43%; 4,259,679) and has a population density of 1,444 inhabitants/km2.^6^ With a nucleus in the capital Recife, the 1^st^ Region is organized into three health micro-regions and has 19 municipalities, in addition to the State District of Fernando de Noronha (Figure 1)^6^.

### Definition of probable cases and data collection

Probable cases of CHIKF correspond to the total number of reported cases, excluding discarded cases that had a negative laboratory diagnosis or that were diagnosed for other diseases, according to the Ministry of Health^19^. Data were obtained from the Notifiable Diseases Information System (*Sistema de Informação de Agravos de Notificação* – SINAN), considering the municipality of residence and epidemiological year of onset of symptoms, made available, in April 2022, by the Epidemiological Surveillance of the 1^st^ Regional Health Management of the state of Pernambuco (*Iᵃ Gerência Regional de Saúde do estado de Pernambuco* – 1^st^ GERES/SES-PE).

### Socioeconomic and demographic variables

The socioeconomic and demographic variables included in the spatial analysis were: Gini Index and Municipal Human Development Index (MHDI), extracted from Atlas Brasil^20,21^; Social Vulnerability Index (SVI), obtained by the Institute of Applied Economic Research (*Instituto de Pesquisa Econômica Aplicada* – IPEA)^22^; and population density (inhabitants/km^2^), provided by the Brazilian Institute of Geography and Statistics (*Instituto Brasileiro de Geografia e Estatística* – IBGE)^23^. The data were from the last Census, carried out in 2010.

All socioeconomic indices used range from zero to one. The MHDI indicates the degree of human development in the municipality related to three subindices — longevity, education, and income —, classified as: very low (<0.500), low (0.500–0.599), medium (0.600–0.699), high (0.700–0.799), and very high (0.800–1)^20^. The Gini index highlights the degree of inequality in the *per capita* household income distribution; the closer to one, the greater the social inequality: low (<0.550), medium (0.550–0.699), high (0.700–0.799), and very high (0.800–1)^21^. SVI expresses the indication of exclusion based on the subindices urban infrastructure, human capital and income and work, being categorized as: very low (<0.200), low (0.201–0.300), medium (0.301–0.400), high (0.401– 0.500), and very high (0.501–1)^22^.

### Aedes aegypti infestation

The vector infestation of the *A. aegypti* mosquito was estimated from the Building Infestation Index (BII), which assesses the risk situation of arbovirus transmission by measuring the population level of the vector when calculating the percentage of properties with the presence of larvae of this Culicidae within the total number of surveyed properties^24^. The calculation is performed using the Rapid Index Survey for *A. aegypti* (*Levantamento Rápido de Índices para A. aegypti* – LIRAa) and the BII is classified as: satisfactory (<1%), alert (1–3.9%), and at risk (>3.9%)^24^. These data were provided by the Environmental Surveillance of 1^st^ GERES/SES-PE.

### Calculation of incidence rates

The outcome of interest in this study was the mean CHIKF incidence rate per municipality, calculated using the ratio between the average of the total number of probable CHIKF cases in each municipality over the 7-year period (2015 to 2021) and the estimated population, multiplied by 100,000 inhabitants, according to Costa et al.^16^. The estimated population for 2018, equivalent to half of the period studied, was obtained from IBGE^23^.

### Spatial analysis

All municipalities were included in the spatial analysis, except for the Fernando de Noronha archipelago, due to different environmental conditions and the difficult visualization of spatial patterns on maps based on their distance. In order to establish the spatial autocorrelation linked only to the average municipal incidence rate of CHIKF, the univariate Global Moran Index was measured. The bivariate Global Moran Index was calculated in order to determine the spatial correlation between this rate and the possible associated factors (population density, Gini Index, HDI, SVI, and BII).

The Moran Global Index evaluates the pattern of spatial distribution and provides a general measure of the degree of spatial association present in the dataset, considering geographic adjacency^25^. It can vary from -1 to +1: indices close to +1 mean that neighboring areas present similar behavior (positive spatial relationship) and indices close to -1 portray that municipalities with low values tend to be close to neighbors with high values and vice versa (negative spatial association). When the index is close to zero, the absence of spatial dependence is revealed due to the random spatial distribution of the data^25^.

From the significant global spatial associations, it was possible to evidence them locally through the graphical representation (LisaMap) of the Local Moran Index and its calculation referring to a specific value for each area^26^. In these representations, clusters of municipalities with significant spatial dependencies are observed. When a municipality has a positive local spatial association with neighbors, it means that both have similar scenarios (high-high or low-low relationship for the value(s) of the variable(s)), analyzing the clusters. In contrast, when municipalities have different conditions (high-low or low-high ratio), outliers are analyzed, which indicate points of negative spatial association^26^.

The indices were estimated using the GeoDa software, version 1.20.0.8. The “first-order” Queen neighborhood matrix was used, involving data from neighbors that touch at least one geographic side of each municipality; and 999 permutations in the pseudosignificance tests, considering a significance level of p<0.05 for spatial relationships. Subsequently, the maps were made using the Quantum Geographic Information (QGis) software, version 3.22.6.

## RESULTS

Between 2015 and 2021, 53,269 probable cases (31,961 confirmed) of CHIKF were reported in residents of the municipalities that make up the Iᵃ RSP, for an average incidence rate of 180.8 cases/100,000 inhabitants over 7 years.

Table 1 shows the number of probable cases by year of onset of symptoms, according to demographic characterization, and it is possible to note that there was a greater involvement of female patients, aged 20 to 39 years and of brown race/color.

**Table 1:** Demographic description of probable cases of Chikungunya fever by year of symptom onset, 1^st^ Health Region of Pernambuco (including Fernando de Noronha), Brazil, 2015–2021. Percentages include discarded/blank data.

		Number of probable cases of Chikungunya fever per year								
		2015	2016	2017	2018	2019	2020	2021	Total	%
		2,606	17,296	973	704	1,497	4,468	25,725	53,269	100
Gender										
	Male	834	6,795	388	259	567	1,729	10,990	21,562	40.5
	Female	1,770	10,482	583	445	928	2,719	14,684	31,611	59.3
Age range (in years)										
	<01	55	333	15	14	30	139	276	862	1.6
	01–04	62	512	21	32	43	93	555	1,318	2.5
	05–09	131	914	52	53	93	149	1,194	2,586	4.8
	10–19	271	1,911	95	84	216	368	2,473	5,418	10.2
	20–39	795	6,064	385	240	514	1,564	9,344	18,906	35.5
	40–59	830	5,009	289	207	411	1,555	8,593	16,894	31.7
	≥60	462	2,549	116	74	189	600	3,268	7,258	13.6
Race/color										
	White	179	605	71	59	167	273	1,807	3,161	5.9
	Black	57	184	19	11	43	96	552	962	1.8
	Yellow	10	23	3	4	5	18	100	163	0.3
	Brown	828	3,138	163	198	554	1,245	9,188	15,314	28.8
	Indigenous	6	22	1	3	6	12	56	106	0.2

Source: SINAN, Iᵃ GERES/SES-PE (April, 2022).

As for the annual incidence rates, 2021 (600.5), 2016 (414.6), and 2020 (104.9) stood out, in contrast to the years 2015 (62.9), 2019 (35.3), 2017 (23.2), and 2018 (16.7), which showed the lowest values.

**Figure 2:**
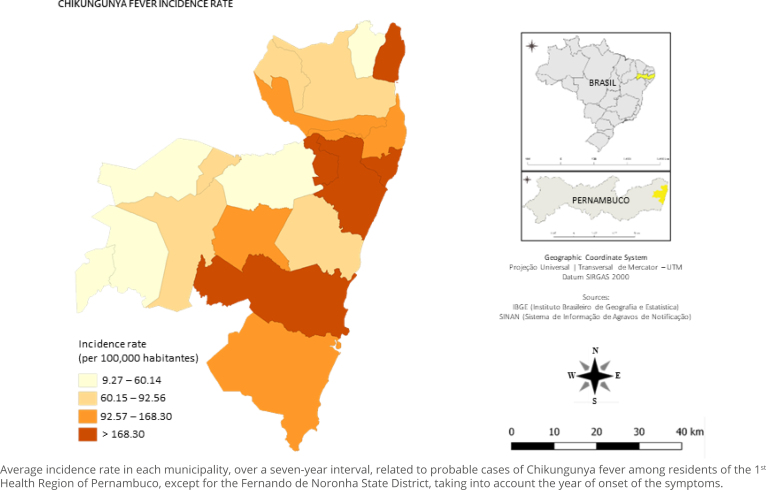
Map with municipalities categorized according to the parameters of the incidence rate of probable cases of Chikungunya fever, 1^st^ Health Region of Pernambuco, Brazil, 2015–2021.

The distribution of municipal incidence rates showed that 10 of the 19 municipalities had high (92.57–168.38) and very high (>168.30) rates: Ilha de Itamaracá (369.92), Cabo de Santo Agostinho (277.41), Recife (262.93), Camaragibe (196.51), Olinda (171.14), Ipojuca (165.92), Moreno (142.44), Abreu e Lima (135.37), and Paulista (108.12). Thus, there was a predominance on the east coast, covering, in sequence, microregions I and III; and a municipality in microregion II, located in the west (Figures 1 and 2).

**Figure 3:**
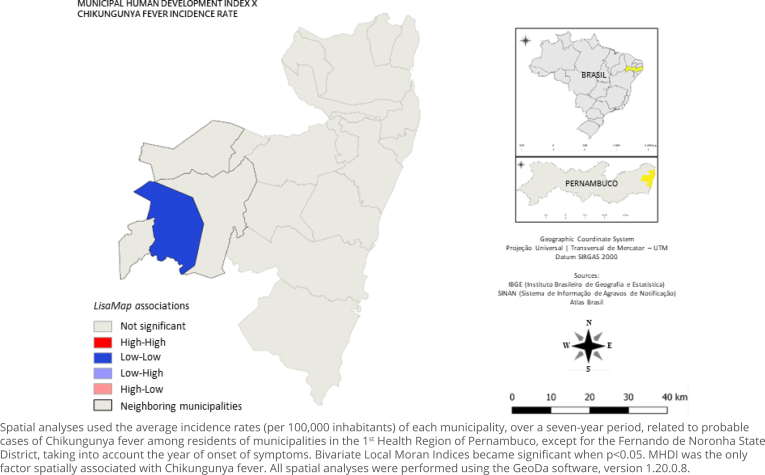
Map of the bivariate local spatial analysis (*LisaMap*) between the incidence rate of probable cases of Chikungunya fever and the Municipal Human Development Index, with its spatial associations, 1^st^ Health Region of Pernambuco, Brazil, 2015–2021.

The Global Moran Index (I) identified a positive spatial autocorrelation for the CHIKF incidence rate, but not significant (I=0.03; p=0.294). However, in the bivariate analyses, there was a significant spatial correlation only between the MHDI and the incidence rate of CHIKF (I=0.245; p=0.038), making the MHDI the only spatially associated factor. The maps elaborated from the analysis of the Local Moran Index showed the nucleus of a “low-low” cluster in Pombos, represented by the dark blue color, portraying that the municipality had low incidence, being surrounded by neighbors that had low values of MHDI (Figure 3). It is noteworthy that population density, the Gini Index, SVI, and BII were non-significant variables and, therefore, factors not associated with CHIKF (Table 2).

**Table 2:** Univariate analysis of the incidence rate of probable cases of Chikungunya fever and bivariate analyses between this rate and demographic, socioeconomic, and environmental variables, 1^st^ Health Region of Pernambuco, Brazil, 2015–2021.

Univariate analysis			
		Global Moran Index	p-value
Incidence rate of probable cases of Chikungunya fever from 2015 to 2021		0.030	0.294

Bivariate analyses with the incidence rate of probable cases of Chikungunya fever from 2015 to 2021			
Characteristics		Global Moran Index	p-value
Demographic			
	Population density	0.189	0.081
Socioeconomic			
	MHDI	0.245	0.038
	Gini index	0.046	0.281
	SVI	−0.128	0.149
Environmental			
	BII	−0.159	0.118

In the bivariate spatial analyses, the average incidence rate of each municipality in the seven-year interval related to probable cases of Chikungunya fever among residents of the 1^st^ Health Region of Pernambuco, except for the Fernando de Noronha State District, was used as the dependent variable. Municipal spatial units were used. Univariate and bivariate Moran Global Indices became significant when p<0.05. MHDI was the only factor spatially associated with Chikungunya fever. All spatial analyses were performed using the GeoDa software, version 1.20.0.8. MHDI: municipal human development index; SVI: social vulnerability index; BII: building infestation index.

## DISCUSSION

This study is the first record to explore a spatial analysis between the incidence of CHIKF and demographic, socioeconomic, and vector infestation indicators in areas of the state of Pernambuco. The univariate analysis identified that the municipalities of 1^st^ HRP did not show spatial autocorrelation for the incidence rates of probable CHIKF cases. In the bivariate analyses, the MHDI revealed a spatial correlation with the disease, showing that municipalities in the west showed spatial dependence between lower MHDI values and lower incidence rates of this arbovirus.

There was a similarity between the demographic characterization of dengue in Pernambuco (2015–2020)^27^ and that evidenced for CHIK in the 1^st^ Region. In the entire study area, CHIKF was present, with a predominance of incidence rates considered high and very high in the Metropolitan Region of Recife (MRR) (microregions I and III); and low and medium in microregion II. However, the spatial distribution of these rates was heterogeneous in the 19 municipalities of the 1^st^ Region, which may have determined the random spatial pattern observed by the Global Moran Index, as found in investigations carried out in the state of Maranhão^16^ and in Barbados^18^, an island country in the Caribbean.

In contrast, studies conducted in the cities of Rio de Janeiro^9^ and São Luís^8^ demonstrated positive and significant global spatial autocorrelation for the incidence rates of suspected and probable cases of this arbovirus, respectively, indicating that adjacent neighborhoods have similar rates. Freitas et al.^28^ showed spatial dependence in municipalities with high incidences of CHIKF in the MRR, Pernambuco, in 2018. The authors, however, did not analyze the possible explanatory factors for the distribution of CHIKF in the state.

In the present spatial analysis, it was found that the incidence rate of CHIKF was spatially directly associated with the MHDI. Consistent with this result, Costa et al.^16^, when investigating CHIKF and Zika between 2015 and 2016, indicated a positive spatial association between the incidence of arboviruses transmitted by *A. aegypti* and the HDI in municipalities in Maranhão. These researchers found relationships between better MHDI values and high incidence rates, suggesting that municipalities with higher MHDI are generally the most populous and have greater access to health services, influencing the quantification of notifications.

Diverging, the current study noted spatial dependence in four municipalities with low incidence rates and low MHDI values in the west, more specifically in microregion II. However, these low incidences may be related to underreporting. With the lowest population densities and lowest levels of development in the entire region, micro-region II probably did not have the infrastructure that would allow greater access to health services^16^ or technical-operational conditions to detect, notify, and investigate cases^29^, thus being able to have cooperated for the lowest number of reported cases. However, the quality of these services was not measured.

The other socioeconomic variables analyzed, the SVI and the Gini Index (social inequality), did not present spatial associations with the incidence rate of CHIKF. Studies with conflicting results about the socioeconomic parameter and the triad of arboviruses (dengue, Zika, and CHIKF) have been recorded: while some indicate the absence of spatial dependence related to the Gini Index and the incidence of these diseases (for dengue^16^), others point to positive (for suspected cases of dengue^15^) or inverse correlations (for CHIKF^16^ and Zika^16^).

The differences between the associations found in different studies may be related to the type of spatial unit used, indicating that secondary data used by the same type of grouping supposedly present similar results^17^. Another issue is that socioeconomic levels are difficult to measure due to the diverse and complex variables involved for aggregate data^17^. Therefore, deeper investigations are needed on the relationship between socioeconomic aspects and CHIKF and other arboviruses, as well as vector infestation.

With regard to demographic data, the spatial dependence analysis did not identify a correlation between population density and the incidence rate of CHIKF. Similar to this result, a survey carried out between 2000 and 2018, in Brazilian municipalities, identified that population density was not spatially associated with the incidence rates of dengue^30^ — an arbovirus transmitted by the same vector as CHIKF. On the other hand, studies suggest that more populated environments favor the spatial diffusion of infectious diseases, especially those transmitted by the *A. aegypti* mosquito^9,16,31^, due to the supply of a high number of individuals susceptible to new infections and the adaptation of the vector to perilous and intradomiciliary environments^9^.

Areas with a high number of inhabitants per square kilometer are usually the result of disorderly urban growth^10^. Together with this, the process of integration of territories with common interests from a “core city”, the so-called metropolization, has been causing several effects to Brazilian cities, such as socioeconomic advances that act, for example, in the MHDI; but also includes social inequality^10^.

The MRR was the most unequal metropolis in Brazil in the fourth quarter of 2020^32^ and represented the majority (63%) of the municipalities in the 1^st^ Region that did not have a Municipal Sanitation Plan or that had a plan under development in 2017^33^. This may have contributed for the high incidence rates of CHIKF found in the area, although there was no spatial association of this rate in the 1^st^ Region.

Precarious sanitation conditions, such as inadequate water supply, sewage, and garbage collection, are also consequences of the intense disorderly urbanization^10^ and are part of the components in the calculation of the SVI^22^. Such characteristics create favorable scenarios for the proliferation of the *A. aegypti* vector, due to the opportunity of standing water reservoirs for the laying of its eggs^7,11^. However, a study carried out in the capital of Rio de Janeiro^9^ showed that the incidence rate of suspected cases of CHIKF showed a positive spatial correlation with variables contrary to these components, indicating that high incidences were spatially related to high percentages of households connected to the public water supply network and adequate sanitation.

With regard to *A. aegypti* infestation, the BII did not obtain a spatial correlation with the CHIKF incidence rate, which is consistent with the analysis conducted in Maranhão^16^ during 2015 and 2016, despite being a contradictory finding. The LIRAa was not performed in some cycles of seven municipalities, mainly in 2020 and 2021, which influenced the measurement of the annual average of the BII and may have contributed to the failure to identify a correlation between this index and the disease incidence rate, in addition to the possibility of deficiency in the execution of the LIRAa also in other years. However, it is possible that the BII does not reflect the reality of the risk of spreading arboviruses in the municipalities, given that the index does not measure the density of *A. aegypti* vector adults, who are responsible for transmitting the virus^34^. New studies are needed to investigate this association and, perhaps, new methodological analyses to predict the risk of transmission of arboviruses, with appreciation and integration of epidemiological and entomological surveillance.

When evaluating notifications of arboviruses in Brazil during the COVID-19 pandemic, Lisboa et al.^35^ found that, from 2019 to 2020 (first pandemic year), there was a reduction in CHIKF cases. However, in the 1^st^ Region, it was possible to notice that CHIKF cases have increased continuously since 2019, especially in the pandemic: compared to the past years, 2020 and 2021 showed, respectively, substantial increases of approximately 298 and 576% in the records. This increase may have been due to the consequences of measures to prevent and control COVID-19^36^, such as home isolation and the reduction in routine home visits by Endemic Disease Control Agents, which are essential for the control of arboviruses^35^. Such actions made it possible, for example, to increase peri- and indoor exposure to *A. aegypti* and to increase waste production, which, added to inadequate disposal, may have favored the proliferation of the CHIKF vector^11^. It should be noted that, even so, it is possible that there is underreporting due, also, to similar clinical manifestations in the early stages of COVID-19^37^ and arboviruses^38^, meaning that the increase in cases verified may be even greater.

In addition to the possibility of an ecological fallacy, which can occur in ecological studies, this research had some limitations. The use of aggregated secondary data to describe large regions such as municipalities may not have captured local variations, thus suggesting the use of smaller spatial analysis units, such as neighborhoods or census tracts^9,17^. There may also have been inconsistencies or delays in completing, quantifying, and processing information in the CHIKF notification forms and in the subsequent entry of data^16^. Another limiting factor is related to the use of data from the 2010 Census for socioeconomic indicators, given that they are the most recent available. The outdated data, together with an alleged underreporting of the disease, may not be showing the real situation of the municipalities.

In view of the above, it is important to train health professionals about notifications and active and continuous investigation of CHIKF cases. Furthermore, the investigation of other socioeconomic, demographic, and environmental variables, as well as climatic aspects^10^, can show the reality of each location and better explain the spatial dynamics of CHIKF, taking as examples: productivity of vector breeding sites, temperature, precipitation, and percentage coverage of the Family Health Strategy. In addition, studies that analyze the simultaneous circulation of arboviruses are necessary in regions of Pernambuco.

Despite the limitations, the study had the following advantages: knowledge of the profile of CHIKF in the 1^st^ HRP since the first autochthonous case registered in the state, showing that high incidence rates were concentrated in the MRR (east), while low rates were concentrated in microregion II (west), where there was possibly underreporting; and the identification of aspects that may be interfering with the incidence rates of CHIKF in the spatial scope of the largest health region in Pernambuco.

Although the only possible explanatory factor for the distribution pattern of CHIKF cases was the MHDI, covering low incidences, it should be noted that socioeconomic, demographic, and environmental variables are important to explain part of the phenomenon and to serve as subsidies to (re)formulation of effective public policy planning and the strengthening of the Unified Health System (*Sistema Único de Saúde* – SUS). The development of public policies aimed at improving indicators would make urban spaces less unequal, better planned and healthier, as well as promoting a better quality of life for residents. In this way, it is expected that there will be a contribution to reducing the transmission of this arbovirus, as well as other diseases, improving collective health.
